# Genetic and environmental drivers of colour and pattern in the Australian jacky dragon (*Amphibolurus muricatus*)

**DOI:** 10.1111/jeb.14066

**Published:** 2022-07-21

**Authors:** Rebecca S. Raynal, Lisa E. Schwanz, Julia L. Riley, Kate D. L. Umbers

**Affiliations:** ^1^ Evolution & Ecology Research Centre, School of Biological, Earth, and Environmental Sciences University of New South Wales Sydney New South Wales Australia; ^2^ Department of Biology Mount Allison University Sackville New Brunswick Canada; ^3^ School of Science Western Sydney University Penrith New South Wales Australia; ^4^ Hawkesbury Institute for the Environment Western Sydney University Penrith New South Wales Australia

**Keywords:** animal model, camouflage, elongation, lizard, maternal effects, phenotype, quantitative genetics, squamate reptile, thermoregulation

## Abstract

The underlying drivers of variation in the colouration (colour and pattern) of animals can be genetic, non‐genetic, or more likely, a combination of both. Understanding the role of heritable genetic elements, as well as non‐genetic factors such as age, habitat or temperature, in shaping colouration can provide insight into the evolution and function of these traits, as well as the speed of response to changing environments. This project examined the genetic and non‐genetic drivers of continuous variation in colouration in a lizard, the jacky dragon (*Amphibolurus muricatus*). We leveraged a large captive experiment that manipulated parental and offspring thermal environment to simultaneously estimate the genetic and non‐genetic drivers of variation in colouration. We found that the overall brightness, the elongation of the longitudinal stripes on the dorsum and the contrast between light and dark patches of the pattern were all heritable. Colouration varied according to the age of the hatchling; however, the thermal environment of neither the parents nor offspring contributed significantly to colouration. It appears that developmental plasticity and maternal effects associated with temperature are not important drivers of variation in our measures of colouration.

## Graphical abstract

Jacky dragons exhibit high variation in dorsal colour and pattern. We investigated the role of heritable genetic and non‐genetic drivers of this variation in colouration. We found only genetic sources contributed to variation in colouration.

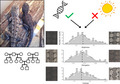

## 1 INTRODUCTION

The colouration of animals is produced via an array of pigmentary and structural mechanisms (Caro, 2005) and can affect evolutionary fitness through communication within and among species, and through thermoregulation. Colouration—the overall combination of colour (hue and chroma), brightness and pattern on the whole body of the animal—can be highly variable within species and be subject to multiple, potentially conflicting selection pressures. For example, potential trade‐offs in brightness (e.g. percentage reflectance) between thermoregulation and camouflage can be important for ectotherms. Most terrestrial ectothermic species bask to reach and maintain active body temperatures (Smith, Cadena, Endler, Kearney, et al., 2016), and the body temperature of animals with darker colouration can increase faster than a paler animal (Bakken & Gates, 1975; Clusella‐Trullas et al., 2009; Smith, Cadena, Endler, Porter, et al., 2016; Watt, 1968). However, a mismatch between animal and background colouration may increase predation risk (Smith, Cadena, Endler, Kearney, et al., 2016). While functions of animal colouration have received considerable attention, the genetic and non‐genetic drivers of variation in animal colouration remain obscured in many cases (Bérénos et al., 2014).

The genetics that underpins colouration are well studied in a few species that have discrete colour polymorphisms in which individuals exhibit one of a few distinct, stable morphs that vary in hue (White & Kemp, 2016). The clearest examples come from work on species in which colour morphs have different behavioural or reproductive strategies (Reviewed in: Cuthill et al., 2017; Olsson et al., 2013). For example, in the side‐botched lizard (*Uta stansburiana*), three co‐occurring colour morphs (blue, yellow and orange) exhibit different male reproductive strategies and arise from a few genes with Mendelian inheritance (Sinervo et al., 2001). Work identifying individual loci driving discrete, adaptive colour variation (Hubbard et al., 2015; Pardo‐Diaz et al., 2015) has focused on dorsal colouration that varies with habitat substrate colour among populations of moths, mice, amphibians and reptiles (Cadena et al., 2017; Hoekstra et al., 2005; Kettlewell, 1961; Krohn & Rosenblum, 2016). For example, in south‐western USA, hair colour in rock pocket mice (*Chaetodipus intermedius*) is darker in populations living on dark‐coloured lava flows than for non‐lava‐dwelling populations, and this variation is controlled by a single locus (Hoekstra et al., 2004). A small number of loci also underpin variation associated with mimetic evolution of wing colour patterns of *Helioconus* butterflies (Martin et al., 2012) and the red and orange ecotypes of monkey flowers (*Mimulus aurantiacus*), which appear to be driving incipient speciation (Streisfeld et al., 2013).

In most species, colouration exists not in discrete polymorphisms but as continuous variation among individuals in a population. Continuous variation in colouration is also likely the product of adaptive evolution in many species, yet its genetic underpinnings have received substantially less research attention particularly within a quantitative genetics framework (San‐Jose & Roulin, 2017). Where it has been examined, additive genetic variance is generally high for continuous variation associated with changes in colour saturation (chroma) or the size of individual colour patches (Table 1). Remarkably, there has been very little examination of heritability in colour patterns despite their ubiquity in nature (Table 1; but see Feiner et al. (2022) for recent genetic markers of colour pattern).

**TABLE 1:** Representative studies investigating the heritability of colouration

Species	Trait	*h* ^2^	References
Great tit (*Parus major*)	Carotenoid content of ventral plumage	~0.03–0.2	Evans and Sheldon (2015)
Barn swallows (*Hirundo rustica erythrogaster*)	Melanin based breast plumage colouration	0.21–0.35	Hubbard et al. (2015)
Barn owl (*Tyto alba*)	Sexually dimorphic melanin and pheomelanin‐based plumage traits	0.57–0.84	Roulin and Jensen (2015)
Atlantic charr (*Salvelinus alpinus*)	Carotenoid content of dorsal skin pigmentation	0.76	Nilsson et al. (2016)
Tawny dragon (*Ctenophores decresii)*	Proportion yellow on throat	0.67	Rankin et al. (2016)
Tawny dragon (*Ctenophores decresii)*	Proportion orange on throat	0.84	Rankin et al. (2016)
Lake Erie island water snake (*Nerodia sipedon)*	Number and height of dorsal and lateral blotches	0.34–0.79	King (1993)
Common gartersnake (*Thamnophis sirtalis)*	Mean dorsolateral blotch pigmentation	0.57–0.79	Westphal and Morgan (2010)
Threespined stickleback (*Gasterosteus aculeatus)*	Intensity (varying from black at 0 to white at 255) of lateral body pigmented area	0.33–0.82	Kim and Velando (2015)
Banana shrimp (*Fenneropenaeus merguiensis*)	Whole‐body colour of raw and cooked shrimp (light or dark)	0.03–0.55	Nguyen et al. (2014)
Orange Sulphur Butterfly (*Colias eurytheme*)	Male dorsal wing colouration, iridescent UV and orange pigment	0.278–0.950	Kemp and Rutowski (2007)

*Note*: For each study, we report the study species, the trait investigated, the heritability index (*h* ^2^) and the reference.

In addition to genetic factors, non‐genetic factors can strongly influence colouration (Olsson et al., 2013). Non‐genetic factors can include those experienced recently by individuals such as diet, body temperature and substrate colour (Norris & Lowe, 1964; Stuart‐Fox et al., 2017; Umbers et al., 2016), those experienced early during ontogeny (Biard et al., 2007; Evans & Sheldon, 2015; Hubbard et al., 2015) and those experienced by their parents (Jensen et al., 2006; Spivak et al., 1990; Tsuruta et al., 1989). Temperature is important through all of these pathways of exposure and can have a long‐lasting influence on colouration (Kooi & Brakefield, 1999). Parental thermal environment has emerged as an important driver of variation in offspring phenotype and fitness, yet its impact on offspring colouration as a parental effect has not been examined (Donelson et al., 2012; Schwanz, 2016; Schwanz et al., 2020; Shama et al., 2014; So & Schwanz, 2018). Thus, given that colouration is important in thermoregulation of ectotherms (Forsman, 1995; Key & Day, 1954; Norris, 1967; Smith, Cadena, Endler, Kearney, et al., 2016; Stuart‐Fox & Moussalli, 2009; Umbers et al., 2013; Watt, 1968) it is possible that temperatures experienced early in life or temperatures that parents are exposed to can lead to long‐lasting changes in colouration and associated thermoregulatory ability (Spivak et al., 1990; Tsuruta et al., 1989).

Understanding the relative importance of non‐genetic factors, particularly temperature, compared with genetics in shaping animal colouration could illuminate the developmental and evolutionary capacity of animals to respond to novel or changing selective pressures. Unfortunately, research on the genetic and non‐genetic drivers of colouration has typically proceeded in nearly separate fields of research, with little integration of these different drivers of variation. However, considering them together is important to gain a full and clear picture of the drivers behind animal colouration (Cadena et al., 2017; Hoekstra, 2006; Olsson et al., 2013). Few studies have partitioned colouration variation into genetic and non‐genetic drivers, and only two (Kemp & Rutowski, 2007; Lewandowski & Boughman, 2008), to our knowledge, have done so while manipulating environmental conditions of potential functional importance. Lewandowski and Boughman (2008) found colour was both genetically heritable and significantly influenced by light environment in benthic threespine sticklebacks, *Gasterosteus aculeatus*. Similarly, in male *Colias eurytheme* butterflies, dorsal wing colouration (iridescent UV and orange pigment) is impacted by nutrition and temperature as well as being heritable (Kemp & Rutowski, 2007). These studies highlight that investigating the effects of environmental variables alongside genetic heritability can be important in understanding the outcome of colouration in many species. Here, we examine variability in colouration by considering potential genetic and non‐genetic drivers together. We studied the jacky dragon, *Amphibolurus muricatus*, a medium‐sized agamid lizard found across the south‐east of Australia (Harlow & Taylor, 2000). Jacky dragons have been used as model species to study temperature‐dependent sex determination (Harlow & Taylor, 2000), maternal effects (Schwanz, 2016) and signalling (e.g. Peters & Evans, 2003). To date, no other study has investigated colour or pattern in jacky dragons.

We used a lab population for which a pedigree was available to estimate the heritability of colouration while simultaneously accounting for the effect of experimental manipulation of parental and offspring thermal environment. We focused on three elements of dorsal colouration: brightness, elongation of dorsal pattern and contrast between the lightest and darkest elements in the pattern. Our hypothesis was that these measures of colouration would be heritable and be influenced by parental and offspring thermal environment. Specifically, we predicted that reduced thermal opportunities in both parental and offspring environmental would be associated with lower brightness, elongation and contrast compared with their experimental counterparts as an adaptive means to absorb greater incident radiation.

## 2 MATERIALS AND METHODS

### 2.1 Study species and housing conditions

The jacky dragon (*Amphibolurus muricatus*) is a small Australian agamid that exhibits substantial variation in colouration (Cogger, 2018). The lizards used in this study were captive‐bred hatchlings sired by wild‐caught and lab‐born parents. The captive colony was collected from Wamboin, Australia (35.25001S, 149.29171E; elevation ∼800 m a. s. l.) and housed indoors at the University of New South Wales. As part of a larger project on parental effects (Schwanz, 2016; Schwanz et al., 2020), parents and hatchlings were maintained under two thermal basking treatments, with offspring treatment randomly assigned (in a split‐clutch design) to match or mismatch their parents' treatments, in a full‐factorial design. The two treatments were long bask (11 h of access to a basking lamp per day) or short bask (7 h of access) conditions, which are ecologically relevant conditions for long day/short day access to thermoregulation (Schwanz, 2016; Schwanz et al., 2020). Data for this study were collected across two consecutive breeding seasons, 2015/2016 (*n* = 155) and 2016/2017 (*n* = 104).

Adult jacky dragons were housed in groups of four during the breeding season: (three females to one male) in opaque plastic enclosures (500 mm × 300 mm × 300 mm) with sand substrate. Each breeding cage was assigned to a basking treatment at the beginning of the breeding season, which could be different for an individual across the 2 years of measurement. The basking lamps created a temperature gradient within the cage (~24–57°C) that allowed individuals to thermoregulate to their preferred temperature while the lamp was on (~35°C; Schwanz et al., 2018). Previous work has shown jacky dragons in both basking treatments remain near their preferred temperature for the majority of the time the lamps are on (Halstead & Schwanz, 2015). Bark, branches and tiles served as shelter and basking objects. Each enclosure lid was aluminium mesh (300 mm × 200 mm) with a 10% UVB light tube adjusted biweekly to match the natural daylight schedule. Adults were fed domestic crickets (*Acheta domestica*) three times a week, with vitamin (calcium and multi‐vitamin) supplementation 1–2 times a week and had *ad libitum* access to water. Females were monitored for gravidity, and most deposited their eggs in nests within their cage. Jacky dragon eggs were collected after deposition and then incubated at a constant temperature of 28°C, which typically produces a 50/50 sex ratio (Schwanz, 2016). As jacky dragons have temperature‐dependent sex determination (TSD), incubation at a constant temperature allows any phenotypic effect arising from sex (male or female) to be de‐confounded from temperature. A week after hatching (the first week is spent in isolation to resorb residual yolk), jacky dragons were placed into either long‐bask or short‐bask treatment conditions themselves. Hatchlings were housed in groups of 1–8 in opaque, white plastic enclosures (250 mm × 300 mm × 300 mm) with white, paper towel substrate. Number of hatchlings within a cage varied in order to split clutches across offspring treatments and to ensure all cagemates were within 1 week of age to each other. Cage and lighting conditions were otherwise the same as for adults. Hatchlings were fed crickets 1 cm in size every day until 1 month old, then five times a week until 3 months old.

### 2.2 Data collection

We quantified three aspects of jacky dragon colouration using digital photography following methods by Troscianko and Stevens (2015) and Endler (2012). Photographs were taken of individual lizard hatchlings from the 2015/2016 season (*n* = 155) and 2016/2017 breeding season (*n* = 104). A total of 259 photographs were taken (one of each jacky dragon). Photographs were discarded if they were not in focus enough to analyse (*n* = 80). We aimed to take photographs of jacky dragons at 30 days old (*n* = 179, mean ± *SD* = 29 ± 11.8 days old). Immediately following photography, individual body surface temperatures were recorded using a non‐contact infrared thermometer.

We photographed jacky dragons in a large cardboard box to prevent escape. The box was lined with a sheet of white PTFE (polytetrafluoroethylene) to standardize the background of each photograph. A Spectralon 99% white reflectance standard (Labsphere, USA; herein referred to as the ‘white standard’) was included in each image to standardize the photograph for analysis. An Iwasaki eyeColour MT70D E27 6500K arc lamp, a broad‐spectrum light source (300–700 nm) with the UV coating removed (sanded lightly with wet/dry sandpaper), was used as the light source for photography (Troscianko & Stevens, 2015). To capture photographs across 400–700 nm, a Nikon DSLR (D90) camera with hot mirror filter removed was used with a JENOPTIK UV–VIS‐IR 60 mm F1.4, quartz optics lens (sensitive to 290–1500 nm). Images were taken of each lizard with a ‘visible’‐pass lens filter to capture 400 to 700 nm wavelengths. Each jacky dragon was placed next to the white standard and photographs were taken in RAW format, using the exposure bracketing function on the camera to ensure at least one image was correctly exposed (Troscianko & Stevens, 2015). The identical set‐up was used for all photographs. Time taken to photograph each lizard once it was placed inside the photography box (2–5 min) was determined by how long it took for them to remain still enough for a sharp photograph to be taken.

### 2.3 Image analysis

**FIGURE 1:**
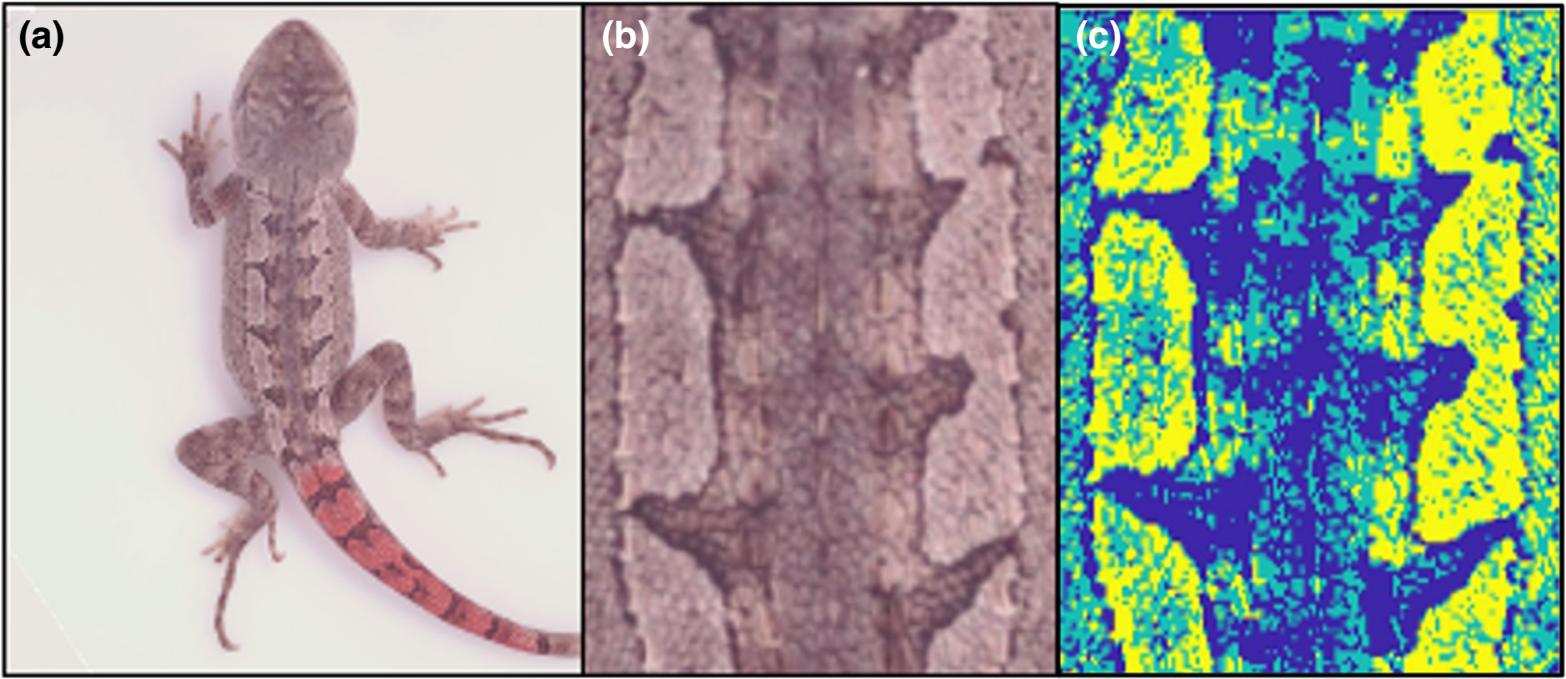
(a) Whole animal photograph focused on its dorsum. The red colouration on the tail was used to temporarily mark lizards for the long‐term study and does not reflect their natural colouration. (b) The corresponding swatch we sampled to quantify their colouration. (c) The artificial colourized representation of how pixels were categorized into colour classes (in this case *k* = 3).

Images of each lizard were white balanced (using the white standard as a reference) and visually inspected for correct exposure using the histograms associated with the images and focus using the photo screening function in the program micaToolbox (Troscianko & Stevens, 2015). The photograph of each lizard was then oriented vertically according to the lizards' longitudinal body axis and cropped into rectangular images ‘swatches’ using the ‘Image J' plugin ‘multispectral imaging’ (Troscianko & Stevens, 2015). Swatches were made by selecting the same area on all individuals: the dorsal area from under the forelegs, over the back, and then extending as far down to the base of the tail as possible without including any pixels of background colour in the swatch (Figure 1).

To quantify colouration, we generated three metrics: ‘brightness’, ‘elongation’ and ‘contrast’ (Endler, 2012). These metrics were chosen from an established set of pattern metrics, many of which have been shown to have functional relevance for antipredator or thermal strategies (Endler, 2012; Rojas et al., 2014; Shine & Madsen, 1994, Trullas et al., 2007). In our study population, the dorsal blotches vary substantially in shape and connectedness (elongation: a measure of separate blotches versus connected stripes) and how bright and distinct they are from the rest of the dorsum colour (contrast and overall brightness). To measure ‘brightness’ for each lizard, we first calculated the brightness of each pixel in the swatch as a value between 0 and 1 (brightness = [R/255 + G/255 + B/255]/3) and then took the mean R, G and B of all pixels as brightness for the whole swatch. ‘Elongation’ and ‘contrast’ (the difference in brightness between the brightness and darkest colour class in the pattern) were generated using the ‘*adjacency*’ function in the ‘*Pavo2*’ package (Maia et al., 2019) in R version 3.5.0 (R Core Team, 2020). This method uses a *k*‐means clustering algorithm to assign pixels to one of a user‐defined number of colour classes (*k*, see also Supplementary Materials; Endler, 2012; Maia et al., 2019). The *adjacency* function surveys each image's pixels at a given grid density to generate metrics that describe the geometry of the pattern and provides the RGB values of each *k*. Measurement of elongation was based on the number of colour class transitions, which are defined as a transition at the pixel level when one colour class changes to another (Endler, 2012). The elongation value is higher if there are fewer up/down (dorsally longitudinal) pixel colour class transitions, than left/right (dorsally transverse) pixel colour class transitions (Figure 1). Contrast was calculated by subtracting the brightness (as calculated above) for the colour class with the lowest brightness from colour class with the highest brightness.

Jacky dragons showed substantial variation in the number of colour classes present to the human observer. To take a rigorous and transparent approach to assigning user‐defined k‐values, we took two approaches and then compared them. First, we assigned the number of colour classes to each individual swatch manually based on human visual assessment (assigned by KDLU and LES; *k* = 2–5) (Table S1). Second, we assigned all swatches the same number of colour classes for both *k* = 3 and *k* = 4. We compared the two approaches statistically using KU visual assignment (Tables S2 and S3). Both elongation and contrast values from manually assigned *k*s were strongly correlated with values calculated when all animals were all assigned *k* = 3 and *k* = 4 colour classes; thus, we felt confident that human manual assignment would not introduce substantial bias to the results (Tables S2 and S3).

To determine whether colour change impacted our measurements, time spent on white background of photography box and body temperature were analysed as potential confounding variables in our study. In addition, to investigate an effect of time on white background, a small experiment was run using five jacky dragons. We found that the time on white background did not influence any of the colouration metrics (see Supplementary Materials for methods and results). To investigate the effect of body temperature, we ran simple linear regressions using the ‘*lm*’ function from the ‘*stats*’ R package (R Core Team, 2020). We found that elongation was impacted by skin temperature (*F* = 8.69, *df* = 1, *p* < 0.01), but contrast (*F* = 0.85, *df* = 1 *p* = 0.36) and brightness (*F* = 0.37, *df* = 1, *p* = 0.55) were not. However, body temperature also differed between study years (*t* = 11.25, *df* = 120, *p* < 0.01, analysed using the ‘*t‐test*’ function in the ‘*stats*’ R package; R Core Team, 2020). Thus, year and body temperature were confounded effects and could not both be included in our models. We opted to include year in our models, as including for the effect of year controlled for both the effect of body temperature as well as differences between researchers and any other unknown variables that differed between study years (see Statistical Methods for more details).

### 2.4 Statistical methods

To estimate the heritability of jacky dragon colouration, we used the animal model (Wilson et al., 2010) with a Bayesian Markov chain Monte Carlo (MCMC) sampling technique in the ‘*MCMCglmm*’ R package (Hadfield, 2010). The animal model is a quantitative genetic mixed‐effect model that includes a pedigree (Wilson et al., 2010). Prior to running the animal model, data were explored to check for outliers, normality of data, and to ensure no collinearity between fixed effects using the data exploration protocol of Zuur et al. (2009). The priors for the regression and variance parameters were: *V* = 1, *n* = 0.002 (Hadfield, 2010). For all models, we estimated parameters 1 500 000 times (iterations) and sampled every 1000th estimation (thinning rate) after the first 1000 iterations were discarded (burn‐in).

We analysed each of the three colouration metrics (brightness, elongation and contrast) separately, using models that contained identical fixed and random effects. All models included the fixed effects of: sex (male or female), parental treatment (long‐bask and short‐bask), offspring treatment (long‐bask and short‐bask), lizard age when photographed (days) and sampling year (2015 and 2016). A previous study with a subset of these lizards found evidence for a three‐way interaction effect between sex, offspring and parental basking treatment on offspring behaviour (McDonald & Schwanz, 2018). So, we also included this three‐way interaction in all our models.

To account for variation due to dependencies in our data and estimate the variance parameters required for calculating the heritability (see below), models also included random effects. Each model included random intercepts for maternal identity (to account for non‐genetic maternal effects), parental cage (to account for cage‐based environmental effects not associated with basking treatment, including non‐genetic paternal effects) and offspring cage (to account for early development conditions not associated with basking treatment). Models also included a random intercept for lizard identity, which linked an individual's data to the pedigree.

In our pedigree, there was a small amount of uncertainty of paternity. A small percentage of hatchlings in our sample (6%) were from females that were housed with different males between seasons, and sperm storage is possible in this species (Olsson et al., 1994, 2009; Rankin et al., 2016; Uller et al., 2013). Here, we used the current male, a female was paired with, as the entire clutch's father. Assuming full‐sib status of offspring in these clutches may, therefore, overestimate relatedness. For our pedigree, we assigned a unique ‘dummy’ father for each wild‐conceived clutch, which assumes full‐sib relationships (multiple paternity in wild jacky dragons is 30%; Warner et al., 2008).

Before interpretation of model outputs and calculation of heritability estimates, model assumptions of normality of residuals and homogeneity of variance were verified (Zuur et al., 2009). We visually inspected all trace plots to ensure they were well mixed. Autocorrelation of the chains of both fixed and random effects was assessed to ensure levels were low (lag < 0.01) using the ‘*autocorr*’ function, and we also performed Geweke and Heidelberg autocorrelation diagnostics (from the R package ‘*coda*’; Plummer et al., 2015). We present the posterior modes and associated 95% credible intervals for each parameter estimate (*β*) and variance (*σ* ^2^) within the models. Parameter estimates were considered significant when 95% credible intervals did not include 0, and the ‘*pMCMC*’ values calculated by ‘*MCMCglmm*’ were <0.05 (Hadfield, 2010).

Variance (*σ* ^2^ *)* estimates from each animal model were used to calculate heritability (*h* ^2^) of their respective colouration metric (brightness, elongation and contrast). The narrow‐sense heritability (*h* ^2^) is defined as the proportion of phenotypic variance ($\sigma_{p}^{2}$) explained by additive genetic variance ($\sigma_{A}^{2}$), which can be estimated using the following formula:

$$h^{2}=\sigma_{A}^{2}/\sigma_{p}^{2}.$$

Phenotypic variance ($\sigma_{p}^{2}$) is the sum of all variance components, including the residual variance ($\sigma_{R}^{2}$), $\sigma_{A}^{2}$, variance (*σ* ^2^) due to mother identity and variance (*σ* ^2^) from offspring and mother housing enclosures (see Table 2 for *σ* ^2^ estimates from all models). Thus, the formula that was used to estimate heritability in this study was as follows:

$$h^{2}=\sigma_{\left(\text{individual}\right)}^{2}/\sigma_{\left(\text{individual}\right)}^{2}+\sigma_{\left(\text{residual}\right)}^{2}+\sigma_{\left(\text{mother identity}\right)}^{2}+\sigma_{\left(\text{individual cage}\right)}^{2}+\sigma_{\left(\text{mother cage}\right)}^{2}$$

**TABLE 2:** Outcome of animal models used to estimate the heritability of jacky dragon (*Amphibolurus muricatus*) colouration

VARIABLES	BRIGHTNESS				ELONGATION				CONTRAST			
Fixed effects	*β*	2.5%	97.5%	pMCMC	*β*	2.5%	97.5%	pMCMC	*β*	2.5%	97.5%	pMCMC
Intercept (2015, female, long‐bask)	**0.3085**	**0.2772**	**0.3467**	**<0.001**	**0.0994**	**0.0647**	**0.1278**	**<0.001**	**0.1382**	**0.1036**	**0.1787**	**<0.001**
Year (2016)	**−0.0345**	**−0.0665**	**−0.0026**	**0.044**	0.0026	−0.0282	0.0206	0.790	**−0.0386**	**−0.0676**	**−0.0076**	**0.010**
Juvenile age at sampling	**0.0017**	**0.0006**	**0.0027**	**0.004**	**0.0014**	**0.0006**	**0.0023**	**<0.001**	**0.0011**	**0.0000**	**0.0020**	**0.060**
Sex (male)	−0.0132	−0.0298	0.0157	0.444	−0.0021	−0.0206	0.0188	0.924	−0.0157	−0.0387	0.0098	0.298
Offspring treatment (short‐bask)	−0.0136	−0.0388	0.0106	0.284	−0.0142	−0.0319	0.0079	0.246	−0.0153	−0.0374	0.0107	0.282
Parental treatment (short‐bask)	0.0088	−0.0272	0.0359	0.772	−0.0022	−0.0224	0.0342	0.674	0.0085	−0.0301	0.0408	0.718
Sex*Offspring treatment	−0.0034	−0.0402	0.0279	0.822	0.0136	−0.0164	0.0405	0.486	−0.0053	−0.0364	0.0316	0.774
Sex*Parental treatment	0.0079	−0.0245	0.0367	0.630	0.0033	−0.0239	0.0319	0.742	0.0118	−0.0221	0.0443	0.524
Offspring treatment*Parental treatment	0.0195	−0.0228	0.0434	0.398	−0.0019	−0.0252	0.0280	0.938	0.0094	−0.0206	0.0445	0.550
Sex*Offspring treatment*Parental treatment	0.0099	−0.0401	0.0485	0.810	−0.0141	−0.0493	0.0317	0.614	0.0096	−0.0381	0.0563	0.748

Random effects	*σ* ^2^	2.5%	97.5%		*σ* ^2^	2.5%	97.5%		*σ* ^2^	2.5%	97.5%	
Juvenile identity	0.0004	0.0002	0.0012	NA	0.0004	0.0001	0.0009	NA	0.0007	0.0002	0.0017	NA
Mother identity	0.0003	0.0001	0.0009	NA	0.0003	0.0001	0.0007	NA	0.0004	0.0002	0.0013	NA
Juvenile's housing enclosure	0.0006	0.0003	0.0013	NA	0.0002	0.0001	0.0006	NA	0.0003	0.0001	0.0006	NA
Mother's housing enclosure	0.0003	0.0001	0.0010	NA	0.0003	0.0001	0.0007	NA	0.0004	0.0002	0.0011	NA
Residual	0.0007	0.0003	0.0011	NA	0.0006	0.0003	0.0008	NA	0.0009	0.0004	0.0013	NA

*Note*: The colouration metrics were quantified for 179 dragons (from 41 offspring cages) from 37 mothers (from 31 parental cages). Our animal models included the fixed effects of year (2015 or 2016), the age at sampling, sex (female or male), offspring and parental thermal treatment (short‐ or long‐bask), as well as an interaction between sex, offspring treatment and parental treatment. They also included the random effects of lizard and mother identity, and offspring and parental cage. We present parameter (*β*) and variance (*σ* ^2^) pooled posterior modes for fixed and random effects, respectively, as well as their associated 95% credible intervals (CIs) and *pMCMC* values. Categorical reference levels for each model variable are supplied in brackets. Bolded values indicate the 95% CIs do not include 0 and the *pMCMC* values were <0.05.

## 3 RESULTS

**FIGURE 2:**
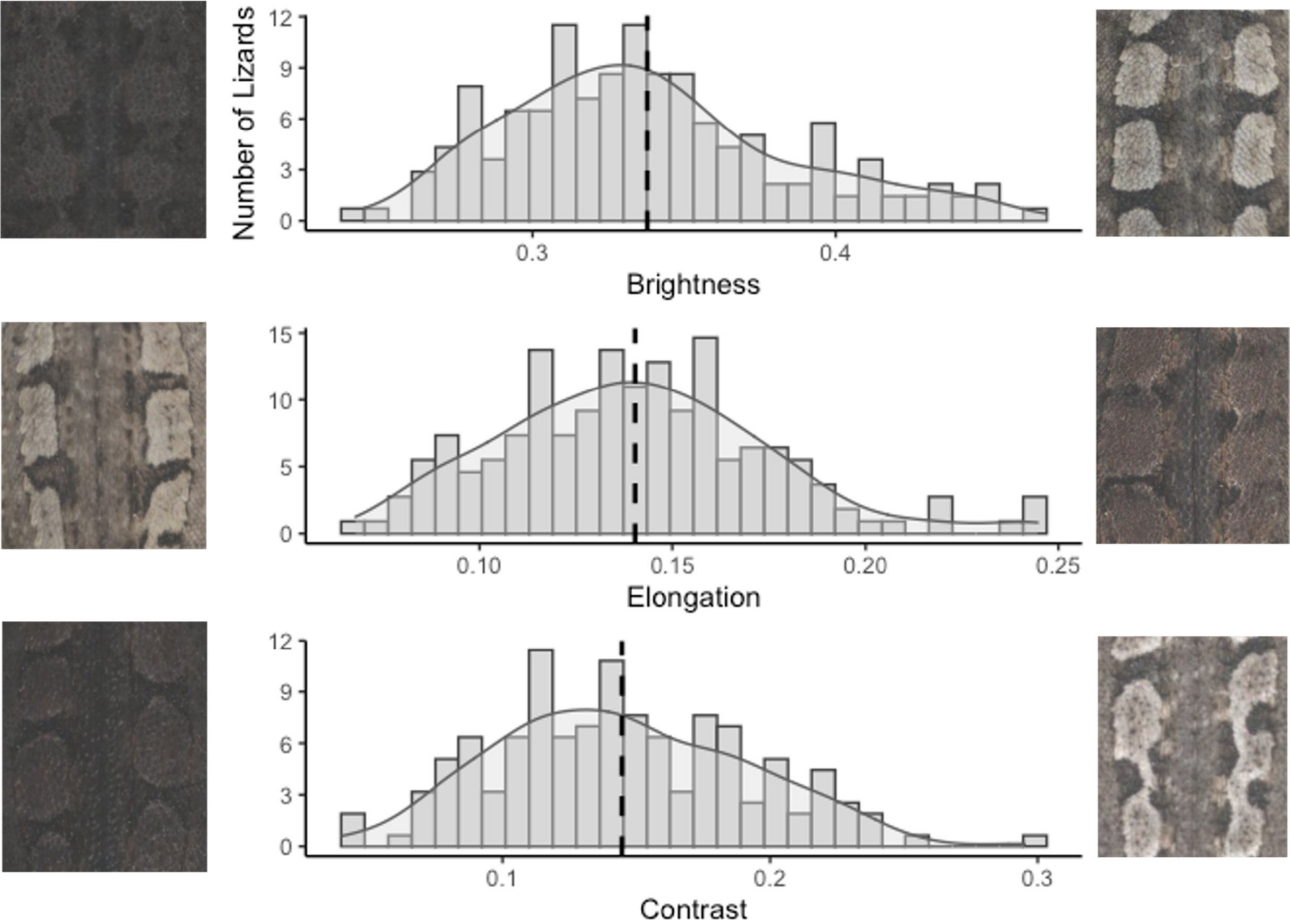
Variation of colouration in jacky dragons that we observed in this study reflected in our three response variables: overall brightness (top), elongation (middle) and contrast (bottom). For each response variable, we present the histogram of values we observed, as well as representative swatches of jacky dragons from the bottom and top range of values of each variable within the study.

We recorded substantial variation across our three colouration metrics, ‘brightness’, ‘elongation’ and ‘contrast’ (Endler, 2012; Figure 2). ‘Brightness’ captured the overall colour brightness across the dorsal image. ‘Elongation’ quantified the connectedness of the longitudinal blotches (i.e. blotches or stripes). ‘Contrast’ captured how well the bright blotches stood out from the dark inter‐blotch background.

**FIGURE 3:**
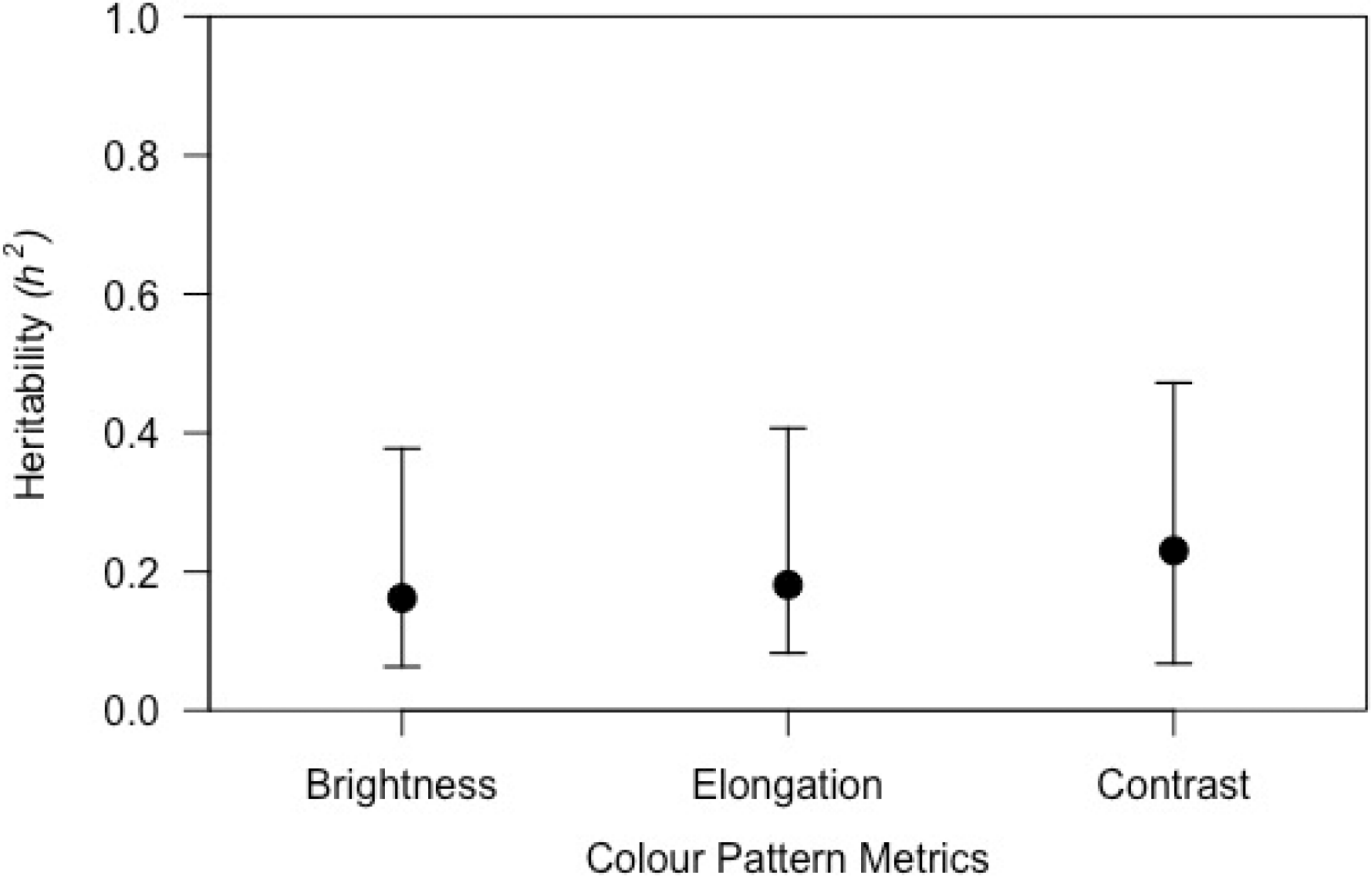
Heritability estimates (*h* ^2^) of colouration metrics: brightness, elongation and contrast depicted with associated 95% credible intervals.

All jacky dragon colouration metrics were heritable (Figure 3). Heritability (*h* ^2^) of brightness was 0.1619 (95% CIs = 0.0630, 0.3774), elongation was 0.1810 (95% CIs = 0.0826, 0.4066), and contrast was 0.2304 (95% CIs = 0.0676, 0.4721). There were also non‐genetic factors that explained significant variation in jacky dragon colouration metrics. Lizard age affected all colouration metrics, as lizards increased in age (days) dorsal colouration became brighter, more elongated and higher in contrast. Sampling year affected two colouration metrics (brightness and contrast); animals in the 2016 sampling year were brighter with higher contrast (Table 2). The two experimental sources of environmental variation—basking treatment in offspring and parents (long‐bask or short‐bask conditions)—did not significantly influence any colour metric. In addition, colouration did not differ between the sex (male or female) of young jacky dragons (Table 2).

## 4 DISCUSSION

Our study suggests that three components of colouration in our captive population of Jacky dragons are heritable: overall brightness, longitudinal stripes on the dorsum (elongation) and degree of difference between light and dark patches of the pattern (contrast). The thermal environment of the parents and offspring that we predicted may have functional importance for these lizards did not significantly influence their colouration. However, more immediate elements of the environment and the individual, as captured in variation across years and animal age, had significant influences on colouration, suggesting that colouration in this lizard is responsive to short‐term conditions.

Interestingly, the levels of heritability of three components of colouration in our population (*h* ^2^ = 0.16–0.23) suggest that colouration could exhibit a robust evolutionary response to selection. Heritability of colouration is often high across a wide array of animal taxa (Table 1). Compared with these studies, our heritability estimates are low (*h* ^2^ = 0.16–0.23). This finding could be attributed to low additive genetic variance or high non‐genetic drivers of phenotypic variance that we could not account for in this study. For example, unmeasured components of individual quality (e.g. resource provisioning and immunocompetence) could be important non‐genetic drivers of variation in jacky lizard colouration, as occurs for many elements of colouration across animals (Pérez‐Rodríguez et al., 2017). However, many of the published colouration heritability estimates come from analysis of either chroma or small colour patches, with a limited number of studies taking into consideration colour and pattern of a whole‐body surface (King, 1993; Westphal & Morgan, 2010; Table 1). The few studies that are similar to ours in examining colouration across large parts of the body (rather than colour patches) similarly report lower heritability estimates. For example, heritability is comparatively low for body colouration (whole‐body colour pattern) in honeybees (*Apis mellifera*; 0.21 for queens and 0.49 for drones; Szabo & Lefkovitch, 1992), ventral body colouration (reflectance) in great tits (*Parus major*; 0.03–0.2; Evans & Sheldon, 2015) and body colour (chroma) in banana shrimp (*Fenneropenaeus indicus*; 0.03–0.55; Nguyen et al., 2014). Therefore, it is possible that whole‐body colouration in general is more likely to have lower heritability than smaller (relative to animal size) patches of colour. The differences in the strength of heritability estimates between colour patches and ‘whole‐body’ colouration raise the possibility that chroma and size of individual colour patches experience different selective pressures than colour patterns, generating differences in standing genetic variance. One possibility is that variation in chroma or patch size tends to be associated with sexual signalling and may thus be driven primarily by directional selection (Olsson et al., 2013), which generally maintains genetic variation. In contrast, if whole‐body colouration is more often associated with non‐sexual functions (e.g. camouflage) and primarily driven by stabilizing selection, then genetic variation would be reduced (Lewandowski & Boughman, 2008; Nguyen et al., 2014; Nilsson et al., 2016; Rankin et al., 2016). Such a hypothesis could only be tested with substantial data across species or focused measures of heritability on components of colouration known to experience different forms of selection within a population.

We found no support for our hypothesis that long‐term thermal environment contributes to variation of colouration in jacky dragons, as neither parental nor offspring thermal environment was significant predictors of colouration. The overall brightness of dorsal colouration in particular has been strongly linked with temperature in other species (Clusella‐Trullas et al., 2009; Kingsolver & Huey, 1998; Rosenblum & Beaupre, 2005). In addition, animal colouration has been shown to change in response to maternal effects (Biard et al., 2007; Tschirren et al., 2012) and environmental effects (Caro, 2005). Our lack of a significant effect of temperature may have several explanations. Firstly, it is possible the selection pressure for camouflage is stronger than thermoregulation. Like many species that live in visually complex habitats, jacky dragons prefer to rest on complex backgrounds (Salisbury & Peters, 2019). Whether or not background choice provides antipredator protection for jacky dragons is unknown; however, birds of prey (Carlile et al., 2006), formidable visual hunters, are important jacky dragon predators that could impose strong selection for detection avoidance through cryptic camouflage. Second, exposure of offspring to a given thermal environment may not have been sufficient to induce a detectable response in colouration (20 ± 1 days exposure; 5% of lizards spent <10 days in their thermal group environment before their photograph was taken). Third, colouration in jacky dragons, or at least the elements that we quantified, may have no functional link to thermoregulation and associated parental effects. While parental thermal environment in jacky dragons alters maternal stress hormone levels as well as offspring post‐hatching growth and size (Liu et al., 2020; Schwanz, 2016; Schwanz et al., 2020), our results suggest that early offspring colouration is not an important component of these parental effects. Lastly, the thermal environment of the parent or young hatchling may not be a good predictor of thermal environment for the rest of the animal's life and thus not be subject to selective pressure within the developmental environment for a particular colouration (Marshall & Uller, 2007). In particular, basking availability changes across days and seasons, so colour change in response to immediate thermal conditions may be more useful than developing fixed differences in colouration early in life.

Whereas our study intentionally minimized variation in three immediate environmental factors (body temperature, light exposure and background) in order to examine other non‐genetic drivers of variation, our significant effects of lizard age and sampling year are likely attributed to uncontrolled ‘immediate’ factors. The effect of lizard age is most likely attributed to shedding cycles differing over the age range at which the photographs were taken (range: 6–62 days). During this time, agamid species can shed their skin 1–2 times depending on individual growth rate (Doneley, 2006). Leading up to a shedding event, jacky dragons' skin will slowly change to a paler, milky appearance, which reduces contrast in their overall colouration, though we would have expected a decline in contrast with age rather than the increase observed. That being said, ontogenetic colour change can be adaptive and is common across invertebrates and vertebrates (Booth, 1990; Medina et al., 2020); thus, exploring how colour differs across ontogeny in this species and linking it to fitness is a potential fruitful direction for future research. The effect of sampling year is most likely due to different researchers taking photographs in each year, perhaps resulting in a slightly different sampling set‐up between the 2 years (e.g. slightly different camera positions). Overall, these effects highlight the importance of short‐term environmental and physiological (non‐genetic) factors influencing variation in the continuous colouration of jacky dragons.

In this study, we examined genetic and non‐genetic drivers of variation in a type of animal colouration that is often neglected in this field—continuous variation in colouration and colour pattern. Moreover, we applied methods for quantifying colour patterns (Endler, 2012) to one of the first analysis of heritability in whole colour patterns. We found that the three components of jacky dragon colouration we assessed—brightness, elongation and contrast—are modestly heritable. Rather than focusing on only the genetic drivers of variation, we leveraged an environmental experiment on a pedigreed population to also examine non‐genetic drivers of variation in colouration. Although we hypothesized that long‐term thermal environment contributes to the variation of colouration in jacky dragons, we found no evidence for this. Future avenues of research could be directed at investigating other possible environmental drivers of variation in colouration, such as complexity of the environment (opportunities for camouflage), short‐term thermal environment and resource availability. In addition, examining the impact of colouration for predator detection, thermoregulation and substrate selection would illuminate the functional significance of this variation. Overall, understanding the genetic and non‐genetic drivers of phenotypic variation can provide a window into the evolutionary past and adaptive potential of colouration.

## AUTHORS' CONTRIBUTIONS

LS and KU conceived the ideas and designed the methodology; RR collected data in 2016/17; RR, JR, KU and LS performed the statistical analysis; RR, LS, KU and JR all contributed to manuscript preparation and approved the submitted version.

## CONFLICT OF INTEREST

The authors declare that they have no conflict of interest.

### PEER REVIEW

The peer review history for this article is available at https://publons.com/publon/10.1111/jeb.14066.

## Supporting information


Appendix S1

## Data Availability

The data used in this study are available on Open Science Framework (OSF) at https://osf.io/c6sy5/.
