# Supplementary material for: Genetic and environmental drivers of colour and pattern in the Australian jacky dragon (Amphibolurus muricatus)
Source: J Evol Biol. 2022 Jul 21;35(9):1229–39. doi: 10.1111/jeb.14066 (PMC9544122; doi:10.1111/jeb.14066)
Supplement: Supplementary file 1 — Appendix S1 [file JEB-35-1229-s001.docx]

Supplementary Materials for

**Genetic and environmental drivers of colour and pattern in the Australian jacky dragon (*Amphibolurus muricatus*).**

Rebecca Raynal, Julia L Riley, Lisa E Schwanz & Kate DL Umbers

**Colour class assignment methods and sensitivity analysis**

Selection of colour classes in generating colour pattern metrics

Colour classes (*k*) can be assigned in two ways. First, a single value of *k* can be chosen based on visual inspection of a selection of colour patterns and then applied it to all lizards, with the software assigning colours to each class. Assigning all lizards a single value of *k* potentially biases the results, if not all lizards have the same number of colours present in their patterns (Fig. S1). Second, the value of *k* can be assigned for each individual lizard on a case-by-case basis based on visual inspection. Assigning *k* to each individual lizard separately is also potentially biased, because it needs to be completed by a human and so is at the mercy of human visual biases, imperfect repetition, imperfect pattern recognition, etc. Given that both these methods are limited, we explored several different scenarios to maximise objectivity and ensure our metrics best represent the lizards’ true patterns.

Human visual colour class assignment

Two researchers (KU and LES) assigned colour classes independently to all 179 photographs. Across all images, both assigned values of *k* between 2 and 5, but only classified *k* with the same value for about half (53%) of all photos.


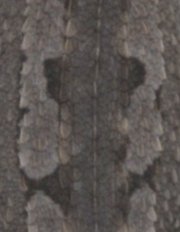

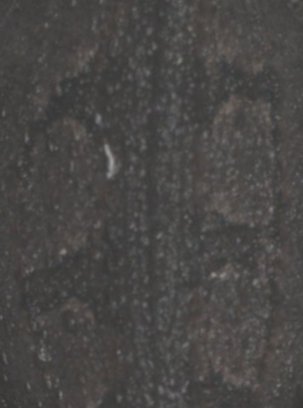


**Figure S1.** Two examples of lizard colour pattern. The lizard on the left (animal 271) was scored as *k* = 4 and the lizard (animal 498) on the right was scored *k* = 2. Colour class representative pixels are marked with a white ring.

Table S1. Contingency table of *k* assignments by two researchers (KDLU and LES) for our 179 photographs. In total, 95 *k* assignments were the same but 84 were different.

|  |  | *k* (LES) | | | |
| --- | --- | --- | --- | --- | --- |
|  |  | 2 | 3 | 4 | 5 |
| *k* (KU) | 2 | 0 | 2 | 1 | 0 |
|  | 3 | 9 | 24 | 15 | 0 |
|  | 4 | 3 | 45 | 71 | 1 |
|  | 5 | 0 | 1 | 7 | 0 |

Uniform assignment of number of colour classes (*k*) to all photographs

The most common *k* values assigned by our two researchers (KU and LES) were 3 and 4. Therefore we compared the colour pattern metrics derived from three different assignments of *k*: applying *k* = 3 to all photos, *k* = 4 to all photos, and KU’s manual assignment of *k*.

Sensitivity of ‘elongation’ and ‘contrast’ to method of colour class (k) assignment

Values of our ‘elongation’ (A [2]; Table S2) and ‘contrast’ (Table S3) colour metrics were highly correlated regardless of the colour class (*k*) assignment method.

**Table S2.** Findings from the regressions between ‘elongation’ colour patterns metric between our three *k* assignments (for all regressions *df* = 1, 179). The top half of the matrix presents *F* statistics and *P* values, and the bottom half presents *adjusted-R^2^* values.

| *k* Assignment | 3 | 4 | KU |
| --- | --- | --- | --- |
| 3 |  | *F* = 2490, *P* < 0.01 | *F* = 2807, *P* < 0.01 |
| 4 | 0.93 |  | *F* = 7645, *P* < 0.01 |
| KU | 0.94 | 0.98 |  |

**Table S3.** Findings from the regressions between ‘contrast’ colour patterns metric between our three *k* assignments (for all regressions *df* = 1, 177). The top half of the matrix presents *F* statistics and *P* values, and the bottom half presents *adjusted-R^2^* values.

| *k* Assignment | 3 | 4 | KU |
| --- | --- | --- | --- |
| 3 |  | *F* > 999, *P* < 0.01 | *F* = 1770, *P* < 0.01 |
| 4 | 0.99 |  | *F* = 2117, *P* < 0.01 |
| KU | 0.91 | 0.92 |  |

**Sensitivity analysis of time spent within the photography box**

To account for the possibility that jacky dragons may change colour in response to light, temperature, or varying time spent on the white background (i.e., background matching), a small experiment was performed on a subset of animals (*n* = 5). Jacky dragons were selected randomly and left in a dark room for 10-20 minutes before their picture was taken, which matched the methodology used within this study’s standard photographic method. The lizard was subsequently moved to the photography box, and a photograph was immediately taken under full spectrum light. Jacky dragons were then left under the light for 5 minutes, which reflected the maximum time a jacky dragon was exposed to the full spectrum light in the standard photographic method. At 5 minutes, a second photo was taken. A total of 10 photos were taken and used in the analysis.

None of the colouration metrics (brightness, elongation, or contrast) significantly differed between individuals immediately photographed after removal from a dark environment vs. the same individual after five minutes spent on the white background in the photography box under full spectrum light (Table S4).

**Table S4**. Outcomes of a linear mixed effects model testing the effect of time in photography box on jacky dragon colour pattern metrics – brightness, elongation, and contrast. This model included random effects of mother identity to account for maternal effects, and animal identity to account for repeated measures of the same jacky dragon (*n*_obs_ = 10; *n*_ind_ = 5; *n*_mumID_ = 3).

|  | *β* | *SE* | *χ^2^* | *P* |
| --- | --- | --- | --- | --- |
| Brightness |  |  |  |  |
| *Intercept* | -0.114 | 0.929 |  |  |
| *Time* | 0.045 | 0.081 | 0.369 | 0.544 |
|  |  | | | |
| Elongation |  |  |  |  |
| *Intercept* | 1.130 | 0.035 |  |  |
| *Time* | 0.004 | 0.004 | 1.220 | 0.269 |
|  |  | | | |
| Contrast |  |  |  |  |
| *Intercept* | 0.293 | 0.029 |  |  |
| *Time* | -0.001 | 0.003 | 0.326 | 0.568 |
